# Diabetes alters vascular mechanotransduction data: Pressure-induced regulation of mTor and associated signaling in the rat inferior vena cava

**DOI:** 10.1016/j.dib.2017.09.001

**Published:** 2017-09-06

**Authors:** Kevin M. Rice, Nandini D.P.K. Manne, Ravikumar Arvapalli, Gautam K. Ginjupalli, Eric R. Blough

**Affiliations:** a Center for Diagnostic Nanosystems, Marshall University, Huntington, WV, United States; b Department of Internal Medicine, Joan C. Edwards School of Medicine, Marshall University, Huntington, WV, United States; c Biotechnology Graduate Program West Virginia State University, Institute, WV, United States; d Department of Health and Human Service, School of Kinesiology, Marshall University, Huntington, WV, United States; e Department of Public Heath, Marshall University, Huntington, WV, United States; f Department of Pharmaceutical Sciences and Research, School of Pharmacy, Marshall University, Huntington, WV, United States; g Department of Pharmacology, Physiology and Toxicology, Joan C. Edwards School of Medicine, Marshall University, Huntington, WV, United States

**Keywords:** Inferior vena cava, Diabetes, mTor, Mechanotransduction, Cardiovascular, Signaling

## Abstract

Diabetes is a multifaceted disease with various etiologies. The complexity of this pathology creates a myriad of factors that must be considered when addressing surgical outcomes and prognosis. Of vital importance to cardiovascular surgery is the viability of homographic vein grafts. Due to the fact, diabetic patients have a higher rate of vein graph failure, a greater understanding of the effect diabetes has on vascular mechano-transductive response is critical to improving patient prognosis. This article represents data regarding a study published in Cardiovascular Diabetology (Rice et al., 2006) [1] and Open Journal of Endocrine and Metabolic Diseases (Rice et al., 2015) [2] with the purpose of evaluating the effect of pressurization on rat inferior venae cavae (IVC). Here we provide the information about the method and processing of raw data related to our prior publish work and Data in Brief articles (Rice et al., Submitted for publication) [3,4]. The data contained in this article evaluates the contribution of mTor signaling and associated proteins. IVC from lean and obese animals were exposed to a 30 min perfusion of 120 mm Hg pressure and evaluated for changes in expression and phosphorylation of mTor, p70s6k, GSK3β, and 4EBP-1.

**Specifications Table**

**Table:** 

Subject area	*Biology*
More specific subject area	*Cardiovascular diabetic surgical tissue response*
Type of data	*graph, figure*
How data was acquired	*immunoblotting*
Data format	*analyzed*
Experimental factors	*IVC mounted vessels were subjected to 120* *mm Hg of pressure for 30 min. Protein was then isolated from tissue for western blot analysis.*
Experimental features	*IVC obtained from Lean and Obese male Zucker rats were used in this experiment*
Data source location	*Data is presented In this article*
Data accessibility	*Data is presented in this article and is related to articles published and in review*[1], [2], [3], [4]

**Value of the data**

• The data presented in this Brief is vital to understanding the effect of diabetes on tissue.
• This data gives insight into the how diabetes alters tissue response to stimuli.
• The data can provide comprehensive analysis of the effect of diabetes on vascular signaling in vein transplant surgery.
• These data provides a more thorough understanding of the mTor involvement in pressure mediated signaling in both diabetic and lean IVC.

## 1 Data

### 1.1 mTor

**Fig. 1:**
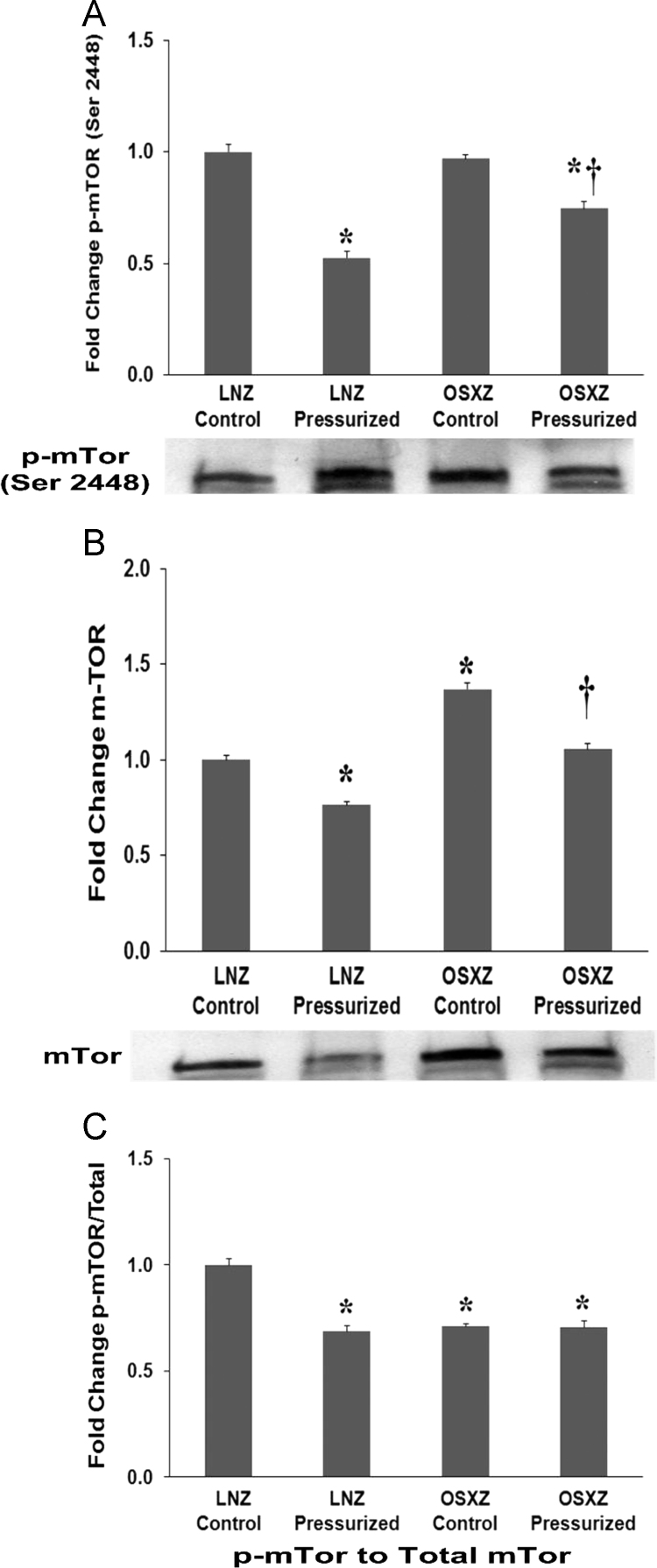
Diabetes alters loading-induced mTor expression and phosphorylation in rat inferior vena cava. The basal (control) and pressure-induced expression of mTor in venae cavae from non-diabetic lean Zucker (LNZ) and diabetic obese syndrome X Zucker (OSXZ) rats. * Significantly different from unloaded venae cavae within the same group (*P*<0.05). † Significantly different from corresponding LNZ venae cavae (*P*<0.05). *n*=6/group.

To determine the effect of pressurization of inferior vena cava (IVC) from diabetic male obese syndrome-X Zucker (OSXZ) diabetic and nondiabetic male normal lean Zucker (LNZ) animals we evaluated the expression of mechanistic target of rapamycin (mTor) [5], [6]. IVCs obtained from the OSXZ control group showed a significant higher level of mTor expression when compared to the LNZ control animals (37±3.0% *p*<0.05). Pressurization resulted in a significant decrease in mTor in the LNZ IVC (24±1.7% *p*<0.05) and OSXZ IVC (31±3.0% *p*<0.05) (Fig. 1-A). Compared to LNZ controls mTor basal phosphorylation at serine 2448 demonstrated no significant difference in the OSXZ IVC. Pressurization of the IVC resulted in a significant decrease in the phosphorylation of mTor in the LNZ IVC (46±2.7% *p*<0.05) and the OSXZ IVC (22±3.1% *p*<0.05) (Fig. 1-B). The ratio of p-mTor to mTOr demonstrated a significant decrease in the basal levels of p-mTor to mTOr in the OSXZ IVC (29±2.2% *p*<0.05) compared to LNZ controls. Pressurization decrease the LNZ (31±3.7% *p*<0.05) ratio of p-mTor to mTOr but failed to change the OSXZ ratio of p-mTor to mTOr, this absence of a pressure induced decrease in the OSXZ IVC was significantly different compared to lean (Fig. 1-C).

### 1.2 p70s6k

**Fig. 2:**
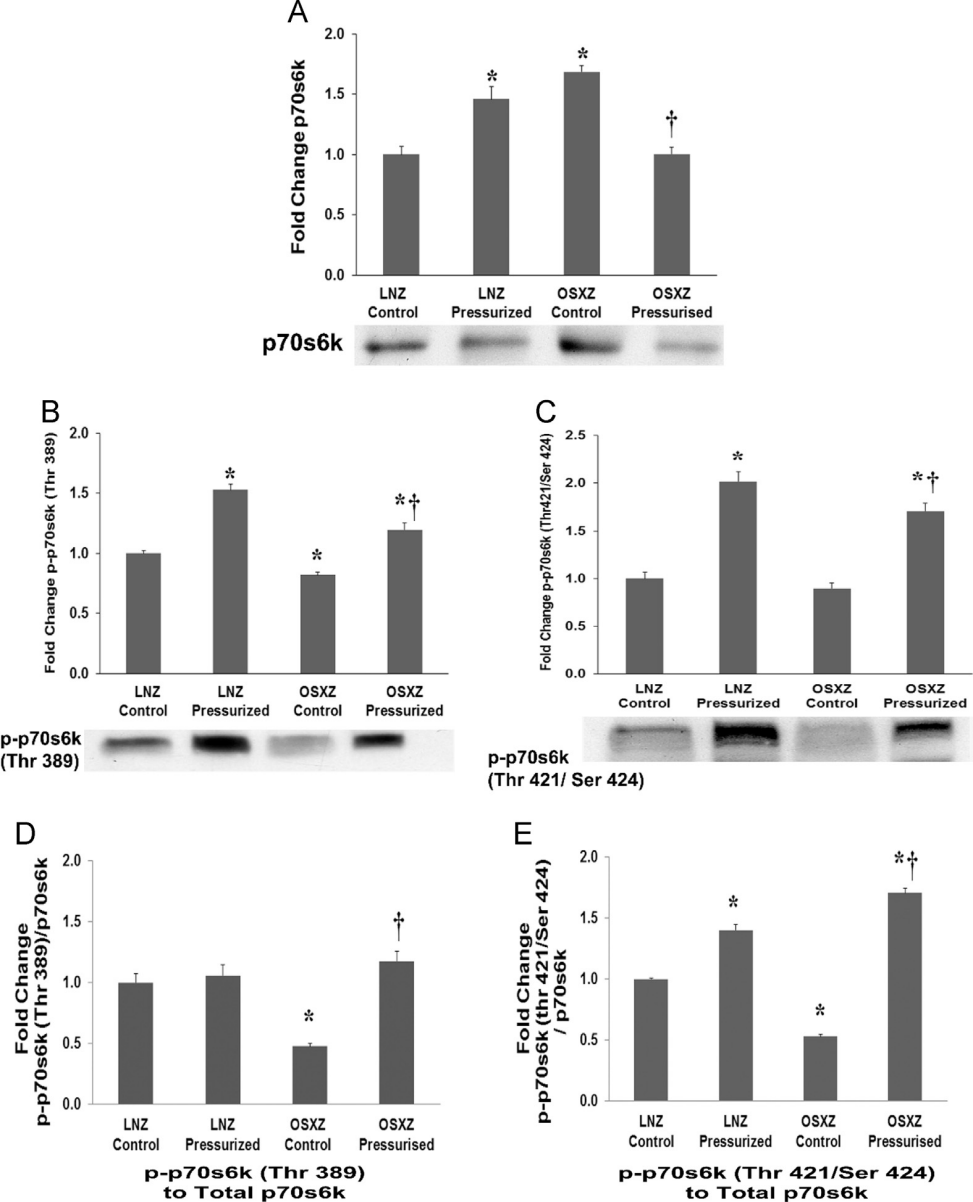
Diabetes alters loading-induced p70s6K expression and phosphorylation in rat inferior vena cava. The basal (control) and pressure-induced expression and phosphorylation of p70s6k in venae cavae from non-diabetic lean Zucker (LNZ) and diabetic obese syndrome X Zucker (OSXZ) rats. * Significantly different from unloaded venae cavae within the same group (*P*<0.05). † Significantly different from corresponding LNZ venae cavae (*P*<0.05). *n*=6/group.

IVCs obtained from the OSXZ control group showed a significant higher level of p70s6k expression when compared to the LNZ control animals (69±5.0% *p*<0.05). Examination of the effect of pressurization of inferior vena cava on p70sk expression demonstrated a significant increase in the LNZ (46±10.6% *p*<0.05) and a significant decrease in the OSXZ (68±6.0% *p*<0.05) (Fig. 2-A). Basal phosphorylation of p70s6k at the threonine 389 (p-p70s6k (Thr 389)) residue demonstrated no significant difference between LNZ and OSXZ. Pressurization increased p70s6k threonine 389 phosphorylation by (53±4.6% *p*<0.05) in the pressurized IVC LNZ animals and (37±5.8% *p*<0.05) in pressurized IVC from OSXZ animals. However the pressure induced elevation in the OSXZ IVC was significantly less than that of the lean (16±9.8% *p*<0.005) (Fig. 2-B). Basal phosphorylation of p70s6k at the threonine 421 and serine 424 residues (p-p70s6k (Thr 421/Ser 424)) demonstrated no significant difference between LNZ and OSXZ. Pressurization significantly increased the phosphorylation of p70s6k at the threonine 421 and serine 424 residues in the LNZ (102±10.2% *p*<0.005) and OSXZ (81±8.2% *p*<0.005) IVC (Fig. 2-C). However, the pressure induced elevation in phosphorylation of p70s6k at the threonine 421 and serine 424 residues in the OSXZ (21±18.3% *p*<0.05) IVC was significantly less compared to LNZ (Fig. 2-C). The ratio of p-p70s6k (Thr 389) to p70s6k demonstrated a significant decrease in the basal levels of p-p70s6k (Thr 389) to p70s6k in the OSXZ IVC (52±9.6% *p*<0.05) compared to LNZ control. No significant changes was detected in p-p70s6k (Thr 389) to p70s6k with pressurization in the LNZ IVC, however, pressurization increase OSXZ (70±8.3% *p*<0.05) ratio of p-p70s6k (Thr 389) to p70s6k (Fig. 2-D). The ratio of p-p70s6k (Thr 421/Ser 424) to p70s6k demonstrated a significant decrease in the basal levels of p-p70s6k (Thr 421/Ser 424) to p70s6k in the OSXZ IVC (47±1.9% *p*<0.05) compared to LNZ controls. Pressurization increase both the LNZ (40±4.7% p<0.05) and OSXZ (117±3.6% *p*<0.05) ratio of p-p70s6k (Thr 421/Ser 424) to p70s6k. However, the pressure induced elevation in the OSXZ IVC was significantly greater compared to lean (78±8.2% *p*<0.05) (Fig. 2-E).

### 1.3 GSK3-beta

**Fig. 3:**
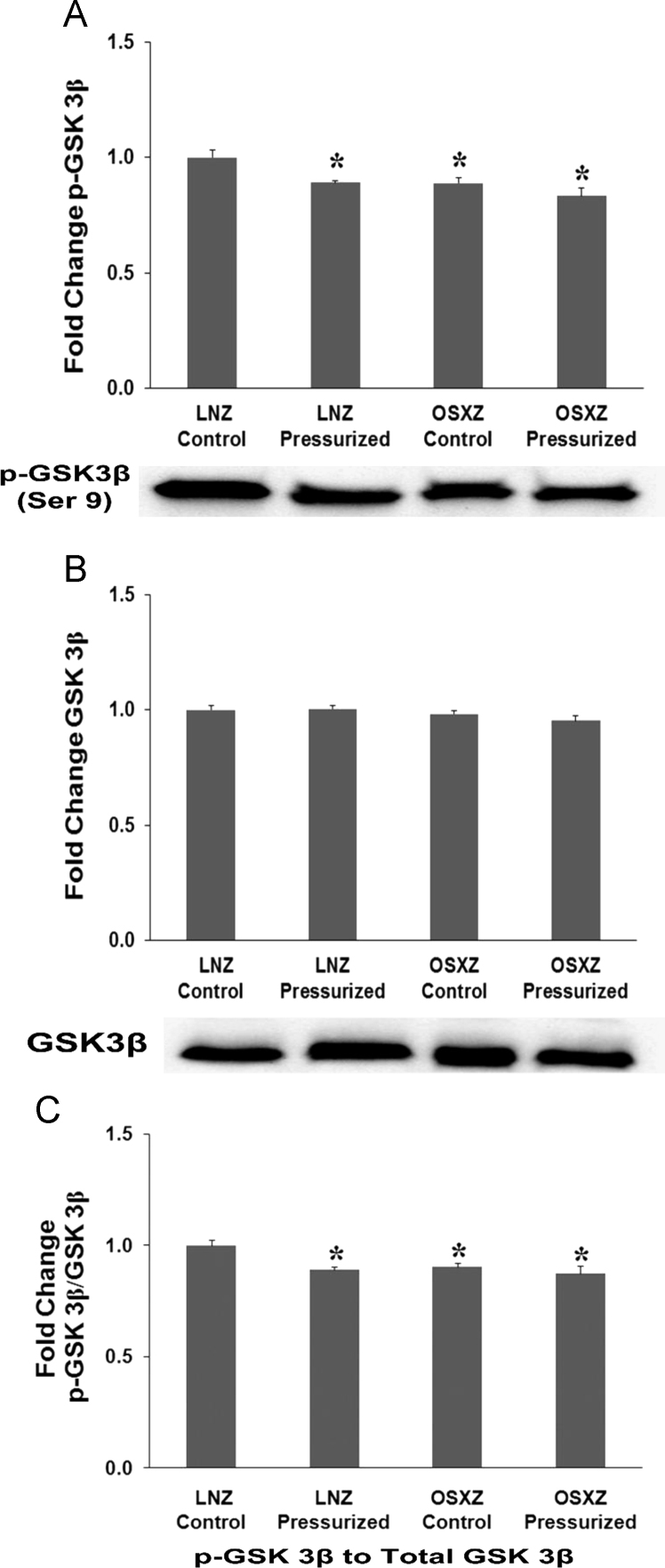
Diabetes alters loading-induced GSK3β expression and phosphorylation in rat inferior vena cava. The basal (control) and pressure-induced phosphorylation of GSK3β in venae cavae from non-diabetic lean Zucker (LNZ) and diabetic obese syndrome X Zucker (OSXZ) rats. * Significantly different from unloaded venae cavae within the same group (*P*<0.05). † Significantly different from corresponding LNZ venae cavae (*P*<0.05). *n*=6/group.

OSXZ IVCs basal GSK-3β levels were not significantly different when compared to LNZ control. Concurrently pressurization resulted in no significant increase in GSK-3β expression in both LNZ and OSXZ IVC (Fig. 3-B) Phosphorylation of GSK-3β demonstrated a slight but significant decrease in the basal levels of p- GSK-3β (Ser 9) in the OSXZ IVC (11±2.5% *p*<0.05) compared to LNZ control. Pressurization of the IVC resulted in a significant decrease in the phosphorylation of p- GSK-3β (Ser 9) in the LNZ IVC (11±0.8% *p*<0.05) but not in the OSXZ IVC (Fig. 3-A). The ratio of p- GSK-3β (Ser 9) to GSK-3β demonstrated a significant decrease in the basal levels of p- GSK-3β (Ser 9) to GSK-3β in the OSXZ IVC (10±1.8% *p*<0.05) compared to LNZ controls. Pressurization decreased the LNZ (11±1.3% *p*<0.05) but not OSXZ ratio of p- GSK-3β (Ser 9) to GSK-3β (Fig. 3-C).

### 1.4 4EBP-1

**Fig. 4:**
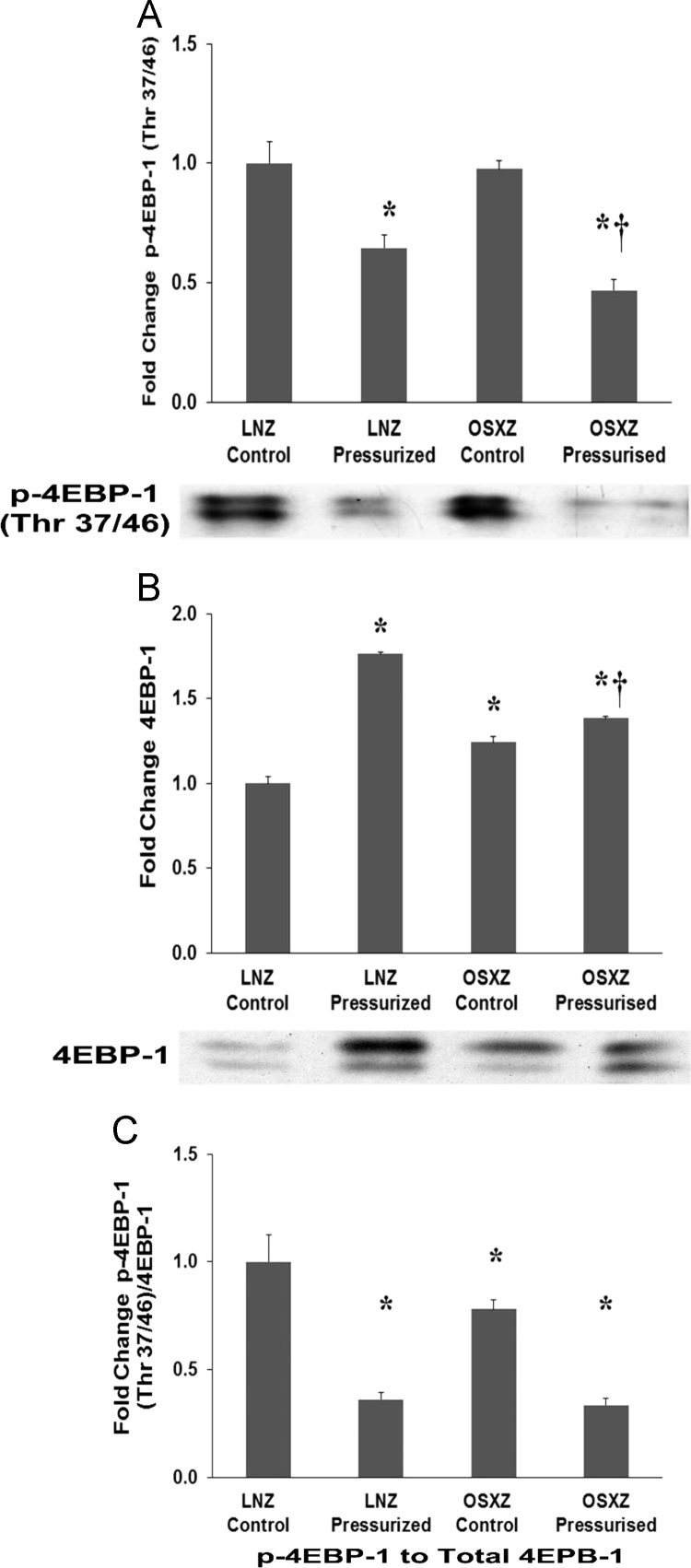
Diabetes alters loading-induced 4EBP-1 expression and phosphorylation in rat inferior vena cava. Phosphorylation of 4E-BP1 results in the dissociation from eIF-4E, allowing the formation of competent eIF-4F complexes [7], [8], [9], [10], [11], [12]. mTOR and mTOR-associated kinase have been shown to phosphorylate 4EBP1 [13]. The basal (control) and pressure-induced expression of 4EBP-1 in venae cavae from non-diabetic lean Zucker (LNZ) and diabetic obese syndrome X Zucker (OSXZ) rats. * Significantly different from unloaded venae cavae within the same group (*P*<0.05). † Significantly different from corresponding LNZ venae cavae (*P*<0.05). *n*=6/group.

OSXZ IVCs basal 4EBP1 levels were significantly higher when compared to LNZ control (24±3.3% *p*<0.05). Pressurization increase 4EBP1 expression in both LNZ (76±1.0% *p*<0.05) and OSXZ (14±1.3% *p*<0.05). However, the pressure induced elevation in the OSXZ IVC was significantly less compared to lean (62±1.3% *p*<0.05) (Fig. 4-B). OSXZ IVCs basal phosphorylation levels of 4EBP1 (Thr 37/46) were not significantly different when compared to LNZ control. Pressurization of the IVC resulted in a significant decrease in the phosphorylation of p- 4EBP1 (Thr 37/46) in the LNZ IVC (36±5.7% *p*<0.05) and in the OSXZ (51±4.3% *p*<0.05) IVC (Fig. 4-A). However, the pressure induced decrease in the OSXZ IVC was significantly greater compared to lean (15±9.9% *p*<0.05) (Fig. 4-A). The ratio of p-4EBP1 (Thr 37/46) to 4EBP1 demonstrated a significant decrease in the basal levels of p-4EBP1 (Thr 37/46) to 4EBP1 in the OSXZ IVC (22±4.4% *p*<0.05) compared to LNZ controls. Pressurization decreased the LNZ (64±3.0% *p*<0.05) and OSXZ (66±3.3% *p*<0.05) ratio of p-4EBP1 (Thr 37/46) to 4EBP1 (Fig. 3-C).

## 2 Experimental design, materials and methods

### 2.1 Animals

All procedures were performed in accordance with the public Health Service policy on animal welfare, Guide for the Care and Use of Laboratory Animals as approved by the Council of the American Physiological Society and the Animal Use Review Board of Marshall University. Rats were obtained from the Charles River Laboratories (Wilmington, MA) (Young (10 week, *n*=12) male normal lean Zucker (LNZ) and young (10 week, *n*=12) male obese syndrome-X Zucker (OSXZ)) and barrier housed one per cage in an AAALAC approved vivarium. Housing conditions included 12H:12H dark-light cycle and temperature was maintained at 22±2 °C. Food and water were provided *ad libitum*.

### 2.2 Materials

Antibodies against p70s6k [cat # 9202], p-p70s6k (Thr421 / Ser424) [cat #9204], p-p70s6k (Thr 389) [cat #9234], mTOR [cat #2972], p-mTOR (Ser 2448) [cat # 2971], GSK 3β [ cat #9338], p-GSK 3β (Ser 21/9) [cat #9331], 4EBP1 [cat #9452], p-4EBP-1 (Thr 37/460 [cat #9459], and p70 S6 Kinase Control Cell Extracts [cat #9203], mouse IgG and rabbit IgG were purchased from Cell Signaling Technology (Beverly, MA)). Precast 10% and 15% SDS-PAGE gels were purchased from Lonza (Rockland, ME). Enhanced chemiluminescence (ECL) western blotting detection reagent was from Amersham Biosciences (Piscataway, NJ). Restore western blot stripping buffer was from Thermo scientific (Rockford, IL). All other chemicals were from Sigma (St. Louis, MO).

### 2.3 Inferior vena cava preparation

Collection, perfusion of the Inferior venae cavae was performed according to the method described by Rice et al. [1], [2], [3], [4]. In brief Rats were anesthetized with a ketamine-xylazine (4:1) cocktail (50 mg/kg ip), the inferior vena cava was isolated aseptically and the *in situ* length was obtained. Vessel were mounted in oxygenated Krebs-Ringer bicarbonate (KRB) buffer maintained at 37 °C and allowed to equilibrate in the vessel chamber for at least one hour before pressure loading. Mounted vessels were subjected to 120 mm Hg of pressure for 30 min to examine the effect of increased loading on signal transduction in the inferior vena cavae.

### 2.4 Immunoblot analysis

After pressurization, Inferior vena cavae were snap-frozen in liquid nitrogen. Protein isolates were prepared according to the protocol described by Rice et al. [1], [2], [3], [4]. In brief samples were pulverized in liquid nitrogen and proteins were extracted using T-PER, diluted to a concentration of 1.5 mg/mL in SDS-loading buffer, ran on 10% or 15% SDS-PAGE gel, transferred onto Hybond nitrocellulose membranes (Amersham Biosciences, Piscataway, NJ) using standard conditions. Membranes were then probed for the molecules of interest using the protocol outlined by Rice et al. [1], [2], [3], [4], and quantified by densitometry using a flatbed scanner (Epson Perfection 3200 PHOTO) and Imaging software (AlphaEaseFC).

### 2.5 Data analysis

Data were analyzed using the Sigma Stat 3.0 and results were presented as mean±SEM. A one-way analysis of variance (ANOVA) was performed for overall comparisons followed by the Student–Newman–Keuls post hoc test to determine differences between groups. The level of significance accepted *a priori* was ≤0.05.
